# Dress Warm

**Published:** 1872-12

**Authors:** 


### DRESS WARM.

WINTER is far more fatal to the old and to invalids, the frail and feeble, than July; this fatality is chiefly in the direction of diseases excited by colds, especially lung affections. Any one of fifty years will be surprised at the number of old friends who have gone before, from pneumonia. It is simply inflammation of the lungs or lung fever; it is a cold settling on the lungs, preceded generally by a slight feeling of chilliness or shiver, running over the body; many times so slight as not to have been noticed. This is mainly the result of our very changeable climate; many times marking a difference of twenty or thirty degrees in a few hours; and when there is a harsh wind, as is often the case, doubling the difference in reality, although not indicated by the thermometer.

The winds are the real source of danger, because they carry the heat from the body with great rapidity. The two most fatal causes are, Having a draught of air on some one part of the body, and Sitting still in a raw, damp place.

It is impossible to be always on the lookout for draughts of air, and in riding, a still position is unavoidable. But there is an easy protection against these, found in warm clothing. The first point is to keep the feet well protected, then the body, especially along the back bone, from the nape of the neck downward, for a breadth of three inches on the line of the shoulders, widening to six inches or more at the small of the back; the want of proper protection there, at all times out of bed, is a fruitful cause of

BRIGHT'S DISEASE

of the kidneys; these important organs of the body were intended by nature to be kept abundantly warm. Hence in health they are enveloped in a case of fat, so as scarcely to be seen, as may be noted any day in a butcher's shop; if chilled, the blood ceases to circulate; it congests, becomes so impacted in the little blood-vessels, that the albumen of the blood is forced through, where only its watery parts were intended to be passed. This albumen is the very life, the support of the body, to give it its strength and flesh; but when it is passed off through the bladder, it is lost, and the man dwindles away to a skeleton; the congestion continuing in some cases, the blood itself follows, and death is inevitable; hence it is specially important in the old, that the small of the back should be comfortably warm.

Between the shoulder-blades behind, the lungs are attached to the body, and at no other point. All know how soon a wind on the back will give a cold. A very few minutes, sitting with the back to a closed window or door, will cause chilliness, even in summer time, because there is a draught of air through the joinings; hence the space between the shoulder-blades should be especially protected in cold weather, either by a strip of buckskin or of stout woolen flannel, attached to the garment ordinarily worn next the skin in the daytime.

But for all over fifty, for the frail and feeble, a better plan is to wear next the skin both drawers and shirt made of very thick knitted or netted material, and made to fit about as close as an ordinary stocking; the action of such a garment is to retain the heat of the body as well as to protect it against the external cold. It has two other advantages: it is open enough to allow the emanations of the body to escape from it, while the actual dampness of perspiration is conveyed from the inner to the outer surface; neither of these advantages can be claimed for perforated buckskin coverings. As a matter of mere experiment, any intelligent reader might try such inner garments for a single winter, and be governed by the result for the future, putting them on about the first of December, not to be removed until June.

